# A proposed method for linear accelerator photon beam steering using EPID

**DOI:** 10.1002/acm2.12419

**Published:** 2018-07-26

**Authors:** Michael P. Barnes, Frederick W. Menk, Bishnu P. Lamichhane, Peter B. Greer

**Affiliations:** ^1^ Department of Radiation Oncology Calvary Mater Hospital Newcastle NSW Australia; ^2^ School of Medical Radiation Sciences University of Newcastle Newcastle NSW Australia; ^3^ School of Mathematical and Physical Sciences University of Newcastle Newcastle NSW Australia

**Keywords:** beam steering, EPID, machine performance check (MPC), pixel sensitivity matrix (PSM)

## Abstract

Beam steering is the process of calibrating the angle and translational position with which a linear accelerator's (linac's) electron beam strikes the x‐ray target with respect to the collimator rotation axis. The shape of the dose profile is highly dependent on accurate beam steering and is essential for ensuring correct delivery of the radiotherapy treatment plan. Traditional methods of beam steering utilize a scanning water tank phantom that makes the process user‐dependent. This study is the first to provide a methodology for both beam angle steering and beam translational position steering based on EPID imaging of the beam and does not require a phantom. Both the EPID‐based beam angle steering and beam translational steering methods described have been validated against IC Profiler measurement. Wide field symmetry agreement was found between the EPID and IC Profiler to within 0.06 ± 0.14% (1 SD) and 0.32 ± 0.11% (1 SD) for flattened and flattening‐filter‐free (FFF) beams, respectively. For a 1.1% change in symmetry measured by IC Profiler the EPID method agreed to within 0.23%. For beam translational position steering, the EPID method agreed with IC Profiler method to within 0.03 ± 0.05 mm (1 SD) at isocenter. The EPID‐based methods presented are quick to perform, simple, accurate and could easily be integrated with the linac, potentially via the MPC application. The methods have the potential to remove user variability and to standardize the process of beam steering throughout the radiotherapy community.

## 1 INTRODUCTION

The process of aligning the angle and translational position with which the linear accelerator (linac) electron beam strikes the x‐ray target with respect to the collimator rotation axis is commonly known as beam steering.^1^, ^2^ The radiation dose profile shape is highly dependent on accurate beam steering and is essential for ensuring correct delivery of the radiotherapy treatment plan. Beam steering is conventionally performed using a scanning water tank. However, this procedure has associated uncertainty^3^ and can be improved upon. The AAPM Task Group 142^4^ recommends that at annual QA testing, any change in the x‐ray beam symmetry from baseline be kept within ±1%. More recently, the AAPM Medical Physics Practice guideline 8.a has relaxed this tolerance to ±2%.^5^

To correctly steer the beam, the user needs to consider beam translational position steering as well as beam angle steering.^1^ The former is often neglected in clinical radiotherapy centers, but both aspects influence the beam focal spot position on the target relative to the flattening filter and collimator axis and hence the resultant dose profile. As such, both alignment processes are necessary.

In the conventional beam steering process, using a scanning water tank, the beam angle and translational position are iteratively adjusted in both planes to optimize the dose profile symmetry. This traditional method has a number of flaws. First, it is by nature iterative since each profile must be scanned individually and sequentially. This precludes real‐time adjustment and makes it difficult to deal with interactions between scan planes and also between angle and position adjustments. Second, the measurement is sensitive to user setup, particularly in leveling the tank. Third, since the tank cannot easily or accurately be rotated with the collimator, then translational positional steering can only be assessed indirectly using beam profile analysis across multiple field sizes. Because of this, the scanning water tank is often used in conjunction with another method for beam translational position steering, such as the half‐beam block. This adds a further complicating process.

These issues have prompted innovation in water tank design, such as inclusion of auto setup capability in an attempt to improve the process. There has also been the development of two‐dimensional (2D) array type devices such as the Sun Nuclear IC Profiler (Sun Nuclear Corporation, Melbourne, FL, USA), which are less expensive than scanning water tanks and allow real‐time adjustment via display of real‐time dose profiles that allow both planes to be viewed simultaneously. Thus, interaction between the two planes can be easily observed and corrected. Such 2D arrays provide potential for significant improvement in beam steering, but potentially can be improved upon further if the linac's onboard electronic portal imaging device (EPID) could be used instead. Using the EPID has a number of potential benefits for beam steering:

1. Since every modern linac has an EPID, then beam steering could be standardized and no extra equipment need be purchased.
2. The highly accurate and repeatable positioning of the EPID could minimize setup variation.
3. The speed at which the EPID can be deployed could reduce setup and measurement time.
4. The 2D nature and small pixel size of the EPID panel provide high resolution and simultaneous measurement in both planes.
5. The EPID can be operated in cine mode to provide real‐time measurement
6. EPID imaging is integrated into the linac functionality. A beam steering application could therefore be produced by the linac vendors to both automate the beam steering process, in particular the image analysis, and also provide a quick routine check of the beam profile and focal spot alignment. This could be achieved by incorporating the methods into subsequent versions of the Varian (Varian Medical Systems, Palo Alto, CA, USA) Machine Performance Check (MPC) application for example.
7. The EPID can be used with new linac designs such as the Varian Halcyon, for which it is more difficult to use a tank because of space limitations.

Varian EPIDs have been used previously for beam profile QA.^6^, ^7^ However, these methods are based on flood field‐corrected EPID images. One of the effects of the flood field is to remove any asymmetry in the beam at the time the flood field is taken. This means that prior to flood field calibration, steering of the beam needs to be performed with an alternate method and subsequent flood field‐corrected EPID measurements can only be used as a constancy check. Hence, flood field‐corrected EPID images are not appropriate for beam steering.

The EPID flood field calibration procedure involves irradiating the whole EPID detector panel. The flood field correction image can be separated into two components: pixel‐to‐pixel sensitivity variation (variation in pixel signal with uniform input), known henceforth as the Pixel Sensitivity Matrix (PSM); and response of the EPID to the nonuniformity of the beam horns (variation in pixel signal with equal sensitivity), known henceforth as the Beam Response. The Beam Response is exaggerated by the EPID compared to an ion chamber measurement due to the increased response of the EPID to low‐energy photons.^8^ The resulting exaggerated beam horns are advantageous for beam symmetry measurement as extra sensitivity is provided.

This study builds on research from different projects for a new application; linac photon beam steering using EPID, which has not previously been attempted. The methods presented have all been modified specific to this new application. Specifically, the work of Yaddanapudi et al.^9^ provides a method of checking photon beam symmetry using PSM‐corrected EPID images for linac acceptance testing purposes and the work of Bin Cai et al.^10^ highlights the advantages of PSM‐corrected EPID imaging for beam profile analysis. However, in our study, we use a different, simplified method of PSM correction, which makes the process more easily adoptable in a clinical setting. We also evaluate the method against the IC Profiler specifically for the beam angle steering application. Secondly, the work of Chojnowski et al.^11^ provides an EPID‐based method for checking linac focal spot alignment with collimator rotation axis, but this method can only be used for beam translational position steering when beam angle steering has been performed immediately prior. In Chojnowski's work, beam angle steering is assumed whereas in our study, we provide an EPID‐based method of checking beam angle steering so that this assumption does not need to be made. We also further evaluate Chojnowski's method against an IC Profiler method. Thirdly, the work of Greer et al.^12^ provides the base theory for determining the PSM (i.e., moving the EPID panel); however, a simplified version of Greer's method is used in this study, which is appropriate to the application and makes the method more easily adoptable.

## 2 METHODS

### 2.A Materials

All measurements in this study were performed on a single Varian TrueBeam 2.5 Stx linac with an aS1200 EPID. The aS1200 EPID utilizes a 43 × 43 cm^2^ panel with a backscatter plate between the detection panel and positioning arm. The detector matrix is 1196 × 1190 pixels providing 0.34 mm resolution at isocenter. The backscatter plate removes EPID arm backscatter as a source of error in the measurements. The principles of this study should be applicable to any EPID panel. However, in this study, the methods have been validated only on the Varian aS1200 EPID and there may be additional considerations for other EPID panel types. For example, with the Varian aS1000 EPID panel, it is necessary to account for EPID arm backscatter that may affect the application of the PSM.

The Sun Nuclear IC Profiler is a 2D ion chamber array specifically designed for beam profile measurements. The IC Profiler utilizes ion chambers separated by 0.51 cm in both the in‐plane and cross‐plane directions, which allows for measurements of up to 32 × 32 cm^2^ field size at isocenter. The IC Profiler has been recently characterized for beam angle steering by Gao et al.^2^ who found agreement between the IC Profiler and water tank wide field symmetry to within 0.7% in 95% of cases measured. However, the study of Gao et al. did not investigate a method of beam translational position steering, although such a method using IC Profiler has been presented by Barnes and Greer^13^ and which is used for comparison in this study.

### 2.B Measurement methods

Beam angle steering can be assessed with dosimetric measurement at a minimum of two equidistant off‐axis beam profile points. The aim of beam angle steering is to achieve equal measured signal at these points (i.e., beam horns of the same height). For beam angle steering in the Varian factory, the field size is set to maximum and the beam is steered using an ion chamber in a fixture that is attached to the collimator in the accessory tray slot.^14^ With collimator rotation, this phantom places the ion chamber at equidistant off‐axis points at 25% and 75% distance across the field. These points minimize the influence of beam translational position steering and jaw positioning on the measurement. In this project, the linac aS1200 EPID is used as the detector for dosimetric measurement of the equidistant off‐axis measurement points. To isolate the Beam Response required to perform the symmetry measurement, the PSM at the measurement points must first be characterized and then removed from a wide field raw EPID image (i.e., not flood field corrected). The beam can then be angle steered so that the Beam Responses at the opposed off‐axis points are equal.

The PSM at the measurement points is measured from EPID images of the same section of the beam that have been imaged with various lateral and longitudinal displacements of the EPID. The concept of imaging with EPID displacement forms the basis of the PSM method of Greer.^12^ The concept is that the PSM can be isolated from the Beam Response by taking a series of images where the beam is kept constant (e.g., 5 × 5 cm^2^ field at central axis), but imaged with different parts of the EPID panel and hence with different PSM. If an image taken with offset EPID is ratioed with an image where the EPID is centered, then the Beam Responses will cancel and the variation from unity in the ratio image will be due solely to differences in the PSM. For the beam steering application, the PSM need only be measured for the off‐axis measurement points, and hence, the complete method of Greer is not required. The procedure for measuring the PSM at these points requires as input the appropriate current EPID flood field, a wide field image and a series of five images, one with EPID centered and a further image each with the EPID panel moved in each of the four directions to the off‐axis measurement points. The flood field is exported from the TrueBeam console and to collect the rest of the data, a plan was created including the six required fields. The first field was the wide field image and the remaining five fields were of the same 5 × 5 cm^2^ field symmetric about central axis, but with different EPID lateral and longitudinal positions that place the off‐axis measurement points on the EPID 10 cm off central axis in the cardinal directions. The plan was created for the 6 MV beam with 100 MU at 600 MU/min dose rate for each field. Copies of the plans were also created but with 10 MV, 6 MV flattening‐filter‐free (FFF), and 10 MV FFF beams and dose rates of 600, 1400, and 2400 MU/min, respectively. Each field was imaged with the EPID in dosimetry mode with the EPID panel at 150 cm SDD. Unfortunately, the TrueBeam linac's inbuilt EPID position rules did not allow the EPID to be moved to +15 cm longitudinally. These rules are also the reason why the measurements were performed at EPID vertical height of 150 cm, as at 100 cm, the EPID positioning rules do not allow lateral and longitudinal motions sufficient to allow measurements to cover enough of the EPID panel for this method. This EPID height is not expected to influence the results.

Once acquired, the images were exported from the Varian ARIA Record and Verify system and subsequently analyzed using a custom Matlab (Mathworks Inc., Natick, MA, USA) script. The script first takes the wide field image and removes the flood field via multiplication to leave the raw whole detector image. Next, the mean values are calculated from a 7 × 7 pixel Region‐Of‐Interest (ROI) at the center of each of the 5 × 5 cm^2^ fields. The mean values are then individually normalized to the field with the EPID panel centered to determine the PSM at each of 10 cm off‐axis positions on the EPID. At each of the off‐axis measurement points, the raw wide field image is then divided by the measured PSM, which results in the Beam Response at each point. The magnitude of the Beam Response at opposing points is then compared as a percentage deviation to provide a measure of wide field symmetry and hence accuracy of beam angle steering.

#### 2.B.1 Region‐Of‐Interest size dependence

A potential weakness of the PSM method is the potential for ROI size to influence the resulting measured Beam Response and PSM. To investigate this, a dataset was chosen and the analysis performed with both the standard ROI size of 7 × 7 pixels (1.6 × 1.6 mm^2^) and a larger ROI of 45 × 45 pixels (10.1 × 10.1 mm^2^). The percentage deviation for each point in the resulting Beam Response arrays was calculated.

#### 2.B.2 Comparison with IC Profiler

##### EPID measured wide field symmetry compared to IC Profiler measured

The PSM at 10 cm off‐axis, henceforth known as the PSM_10_, was measured for each available photon beam energy (6 MV, 10 MV, 6 MV FFF and 10 MV FFF) and then used to determine the Beam Response from wide field raw images. Symmetry was calculated for each beam in both in‐plane and cross‐plane. The IC Profiler was then used to measure the dose profiles at d_max_ for the same beams using a 30 × 30 cm^2^ field size. In document 60976, the International Electrotechnical Commission (IEC) defines beam symmetry as the maximum ratio of the higher to lower absorbed dose at any two positions symmetrical to the radiation beam axis and inside the flattened area.^15^ The IEC definition of symmetry was used in this study to compare the EPID to IC Profiler results.

##### EPID measured symmetry sensitivity to beam angle steering

To test the sensitivity of the Beam Response to changes in beam angle steering, the EPID method was performed pre‐ and post beam angle steering of the 6 MV beam. The change in EPID measured symmetry was compared with the change in symmetry as measured by the IC Profiler.

### 2.C Translational beam position steering using EPID compared to IC Profiler

The methodology presented so far only applies to beam angle steering. However, to steer the beam correctly, a methodology is also required for beam translational position steering. If beam angle has been correctly steered, then misalignment of the focal spot with collimator axis is due to misaligned translational position steering. As such, once beam angle steering has been achieved using the methods already presented, then the method of Chojnowski et al.^11^ can be used to align the focal spot via beam translational position steering. The method is both EPID‐based and phantomless and hence would fit in well with the PSM_10_ method for beam angle steering in a phantomless EPID‐based beam steering application. For this purpose, the method of Chojnowski was further validated by comparison to the departmental method of focal spot alignment and hence translational position steering based on the IC Profiler.

The departmental method of focal spot alignment using IC Profiler was presented in Barnes and Greer.^13^ In this method, the focal spot alignment to collimator rotation axis is determined using the IC Profiler beam center parameter. 10 × 10 cm^2^ fields are measured with the Profiler in the gantry mount at both collimator 90 and 270 degrees. In the gantry mount, the Profiler rotates with the collimator; hence, the sense of the collimator 270 degree beam center measurement was reversed to put both beam center measurements in the same coordinate system. The mean beam center between collimator 90 and 270 measurements then represents the distance of the focal spot from center of collimator rotation. This method is independent of jaw position and IC Profiler positioning. Focal spot position measurements were performed using both the IC Profiler method and EPID‐based method of Chojnowski et al. with 6 and 18 MV beams and results were compared.

### 2.D EPID panel position reproducibility

The accuracy and reproducibility of the EPID panel positioning are essential for the reproducibility of the proposed EPID beam angle steering method. The setup uncertainty of the method will be dependent on how well the EPID reproduces its vertical, lateral, and longitudinal positions and the ability to level the panel when the EPID is deployed. The leveling of the panel can easily be checked either immediately before beam steering or routinely using a spirit level placed on the panel. At the same time, the vertical position can be checked with a front pointer or tape measure. The EPID panel lateral and longitudinal position reproducibility can be checked as part of the proposed beam translational position steering measurements. Once the focal spot has been correctly aligned using the Chojnowski method, then the position of collimator axis on the EPID panel can be measured in both lateral and longitudinal directions and compared to the position measured at PSM_10_ calibration. The long‐term reproducibility of the EPID panel lateral and longitudinal positions was assessed this way using the 2 × 180 degree collimator opposed jaw‐defined fields from the Chojnowski method. From EPID images of these fields, the 50% penumbra positions were measured on the EPID and the midpoint calculated in both lateral and longitudinal directions to subpixel resolution using cubic spline interpolation. The calculated midpoints, hence known as the Central Axis (CAX), were averaged between the collimator opposed fields to remove the effect of any jaw miscalibration. The results were recorded monthly for a period of 2 yr.

## 3 RESULTS

### 3.A ROI size dependence

The results of Table 1 show the variation in measured beam response at each of the measurement points when PSM_10_ was calculated using a 7 × 7 pixel ROI compared to a 45 × 45 pixel ROI on the same dataset. The mean deviation was 0.14 ± 0.06% (1 SD).

**Table 1:** Measured beam response dependence on ROI size

EPID position (mm)		Beam response		% deviation
Lateral	Longitudinal	7 × 7 ROI	45 × 45 ROI	
0	100	1.077	1.075	0.19
0	−100	1.082	1.080	0.19
100	0	1.079	1.078	0.09
−100	0	1.075	1.074	0.09

### 3.B EPID measured wide field symmetry compared to IC Profiler measured

Table 2 compares the measured wide field symmetry between the EPID method and IC Profiler. The mean percentage difference between EPID and Profiler was measured at 0.19 ± 0.18% (1 SD).

**Table 2:** Wide field IEC symmetry as measured with EPID and IC Profiler for all four available photon beams

Beam	Plane	IC Profiler symmetry (%)	EPID symmetry (%)	% difference
6 MV	In‐plane	100.4	100.46	−0.06
	Cross‐plane	100.3	100.34	−0.04
10 MV	In‐plane	100.6	100.38	0.22
	Cross‐plane	100.5	100.38	0.12
6 MV FFF	In‐plane	100.4	100.23	0.17
	Cross‐plane	100.4	100.00	0.40
10 MV FFF	In‐plane	100.7	100.40	0.30
	Cross‐plane	100.4	100.00	0.30
		Mean difference		0.19 ± 0.18% (1 SD)

### 3.C EPID measured symmetry sensitivity to beam angle steering

Table 3 shows the measured symmetry for the 6 MV beam before and after beam angle steering as well as the measured change with the IC Profiler and EPID methods. The cross‐plane direction had the greatest measured change in symmetry at 1.10% using Profiler and 0.87% using EPID.

**Table 3:** Sensitivity of EPID measured wide field IEC symmetry to beam angle steering of the 6 MV beam

	Plane	IC Profiler symmetry (%)	EPID symmetry (%)	% difference
Before steering	In‐plane	101.0	100.88	0.12
	Cross‐plane	101.4	101.21	0.19
After steering	In‐plane	100.4	100.46	−0.06
	Cross‐plane	100.3	100.34	−0.04
Measured change	In‐plane	0.6	0.42	0.18
	Cross‐plane	1.10	0.87	0.23

### 3.D Translational beam position steering using EPID compared to IC Profiler

Table 4 shows the measured focal spot/beam translational steering misalignment at isocenter as measured with both the EPID method and IC Profiler method at two different beam energies. The mean agreement between the two methods was −0.03 ± 0.05 mm (1 SD). This indicates agreement between the two methods within one standard deviation.

**Table 4:** Measured focal spot misalignment/beam translational position steering error at isocenter as measured with both EPID and IC Profiler

Beam	Plane	IC Profiler focal spot position error (mm @ isocenter)	EPID focal spot position error (mm @ isocenter)	Difference (mm) (Profiler – EPID)
6 MV	In‐plane	0.05	0.02	0.03
	Cross‐plane	0.0	0.09	−0.09
18 MV	In‐plane	0.0	0.06	−0.06
	Cross‐plane	−0.05	−0.04	−0.01

### 3.E EPID panel position reproducibility

**Figure 1:**
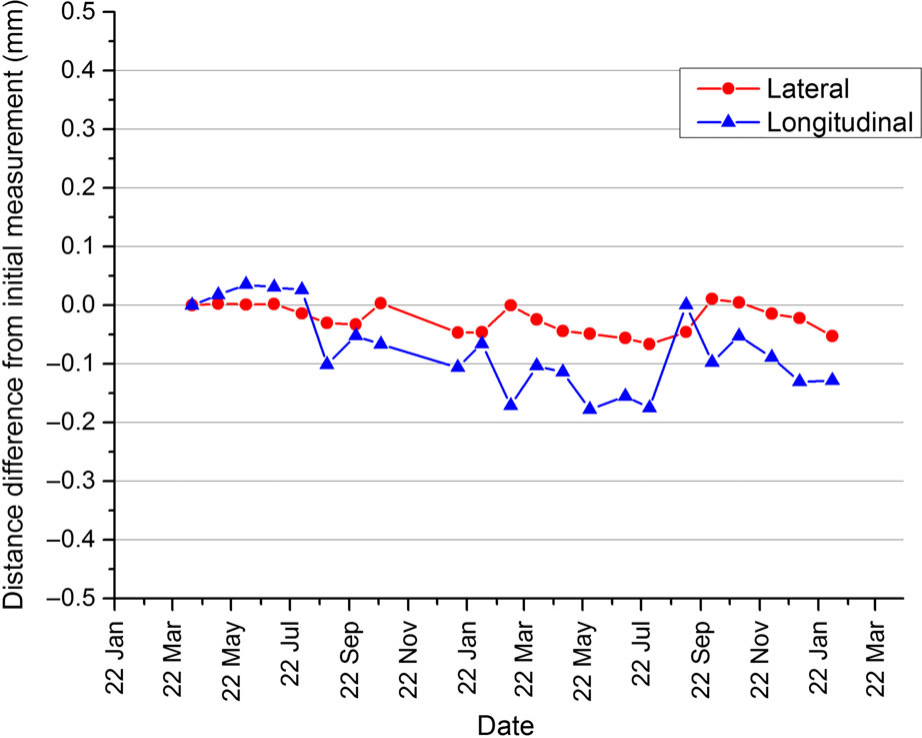
EPID panel lateral and longitudinal position reproducibility over a 2‐yr period.

Figure 1 shows the measured EPID CAX positions over the 2‐yr period for both lateral and longitudinal directions. As stability rather than absolute position are important in this study, the CAX results are presented in Fig. 1 as measured distance (mm) change from the baseline. In the lateral direction, the mean measured distance change is 0.024 ± 0.024 mm (1 SD) and 0.076 ± 0.070 mm (1 SD) in the longitudinal direction.

## 4 DISCUSSION

### 4.A Region‐Of‐Interest size dependence

The results of Table 1 show that the Beam Response measurement is within 0.2% for a ROI size change from 7 × 7 pixels to 45 × 45 pixels. The change is symmetric about the central axis in both planes which indicates a change in the shape of the Beam Response. Since this variation is symmetric, then the wide field symmetry measurement will not be influenced.

### 4.B EPID measured wide field symmetry compared to IC Profiler measured

The results of Table 2 show agreement in symmetry between the EPID method and IC Profiler to within 0.19 ± 0.18% (1 SD) across all four photon beams and both measurement planes. For conventional water tank methods, achieving wide field symmetry of 1% is often deemed acceptable.^4^

Table 2 shows greater discrepancy in wide field symmetry measurement between EPID and IC Profiler for FFF beams compared to flattened beams. The mean FFF disagreement is 0.32% while the mean flattened beam disagreement is 0.06%. This is likely due to the greater dose gradient at the measurement points for the FFF beam compared to the flattened beam. This gradient means that setup errors in both IC Profiler and EPID will have an exaggerated influence. Varian FFF beams utilize the same electron beam (including beam steering settings) as the equivalent energy flattened beam.^16^ Treatment centers whose beams are configured in this way could simply beam angle steer the flattened beam and then set the same steering settings for the corresponding FFF beam.

### 4.C EPID panel position reproducibility

The EPID panel positioning results of Fig. 1 shows subpixel and hence submillimeter reproducibility, which are clinically insignificant. However, the panel positioning reproducibility will be machine dependent and may vary over time. As such, this should be checked as part of the routine linac QA program with tolerances applied the same as that are applied to water tank detector positioning.^3^

### 4.D Proposed workflow

To ensure ongoing accurate beam steering, it is proposed that both the EPID‐based wide field symmetry and focal spot alignment tests be performed as a check on a monthly basis (daily if incorporated into MPC). The results presented here show that these two tests will ensure both accurate beam angle and also beam translational position steering and hence a symmetric beam in accordance with TG‐142 recommendations. It should be achievable for vendors to implement these methods in an integrated application such as MPC. For adjustment of beam steering, the methods of this study could also potentially be incorporated into an automated application. In such an application, it is envisaged that the PSM_10_ measurement be first checked. Next, a wide field open image would be run in continuous acquisition mode with the newly measured PSM_10_ applied. If symmetry is calculated on the images in real time, then beam angle steering can be performed in both planes simultaneously. Alternatively, it may be possible for vendors to calibrate the measured asymmetry to a required change in the linac's steering coil currents. The adjustment can then simply be made and then verified. Once beam angle steering has been adjusted so that the beam horns are at equal height at the off‐axis points, then the series of four fields required for focal spot alignment would be run and translational position steering iteratively adjusted until there is no misalignment. Again, it may be possible for vendors to calibrate the focal spot misalignment to the required change in steering coil current. Once translational position steering is completed, a final wide field symmetry measurement is performed to ensure that any adjustment to translational position steering has not compromised the angle steering. If desired, the user could confirm that the final beam profiles are acceptable using independent measurement.

### 4.E General discussion

The proposed methodology may offer significant time savings if the image analysis can be automated by the vendor. Another advantage is consistency of setup and standardization of both the detector and the approach to linac photon beam steering. Varian has demonstrated the capability to automate EPID image analysis for QA in the MPC application. The reproducibility of the EPID panel positioning has been demonstrated for the linac used in this study and the methods of checking the EPID setup are straightforward. In the case of the EPID lateral and longitudinal positioning, this check is embedded in the method. The potential influence of dead pixels is a consideration for PSM measurement. The effect of a dead pixel would be greater with smaller ROI size, but the chance of a dead pixel in the measurement is more likely in a larger ROI size; hence, it is harder to predict which ROI size would be superior overall. However, ensuring an up‐to‐date dead pixel map is straightforward and is the simple solution for mitigating the risk. For consistent PSM measurement, the lateral and longitudinal motions of the EPID panel must be reproducible. It is proposed to check these routinely with a ruler placed on the panel referenced against a laser.

A weakness in the current study is the need to use off‐axis points only 10 cm from central axis. This is required because of the limitation in allowed EPID panel movement in the longitudinal direction for this linac type, which limits where the PSM measurement can occur. To minimize the influence of translational positional beam steering on the beam angle steering, it is also preferable to measure at 15 cm off‐axis rather than 10 cm. Currently, the Sun Nuclear Daily QA3 device, which is commonly used for daily checks of beam symmetry, utilizes a 20 × 20 cm^2^ field and symmetry assessment on two off‐axis points 8 cm either side of central axis. This is comparable to the EPID measurements at 10 cm off‐axis presented in this study.

A further weakness of the methods is that they currently only apply with the Varian aS1200 EPID. This is because of the backscatter plate, which is not included on earlier Varian EPID models and which removes EPID arm backscatter that influences the PSM measurement. All new TrueBeam linacs are now released with the aS1200 EPID and the problem could be addressed with older EPID models by correcting all images with backscatter correction models using the method of Rowshanfarzad et al.^17^ or similar.

## 5 CONCLUSIONS

Conventional methods for linac beam steering are user‐dependent and non‐standardized. Improvements in this process should be welcomed by all radiotherapy departments. The accuracy of the methods proposed in this study have been demonstrated on a Varian aS1200 EPID with comparison to IC Profiler. With inclusion of automated image analysis by the linac vendor, beam steering verification and adjustment could be performed quickly and consistently with comparable or improved accuracy compared to conventional methods.

## CONFLICT OF INTEREST

The authors declare that they have no conflict of interest in this study.
